# Daratumumab Prevents Experimental Xenogeneic Graft-Versus-Host Disease by Skewing Proportions of T Cell Functional Subsets and Inhibiting T Cell Activation and Migration

**DOI:** 10.3389/fimmu.2021.785774

**Published:** 2021-12-20

**Authors:** Yang Gao, Wei Shan, Tianning Gu, Jie Zhang, Yibo Wu, Xiaoqing Li, Xiangjun Zeng, Hongyu Zhou, Zhi Chen, Haowen Xiao

**Affiliations:** ^1^ Department of Hematology, Sir Run Run Shaw Hospital, Zhejiang University School of Medicine, Hangzhou, China; ^2^ Bone Marrow Transplantation Center, The First Affiliated Hospital, Zhejiang University School of Medicine, Hangzhou, China; ^3^ Liangzhu Laboratory, Zhejiang University Medical Center, Hangzhou, China; ^4^ Institute of Hematology, Zhejiang University, Hangzhou, China; ^5^ Zhejiang Province Engineering Laboratory for Stem Cell and Immunity Therapy, Hangzhou, China

**Keywords:** daratumumab, CD38, graft-versus-host disease, graft-versus-leukemia, chemokine, chemoattractant receptor

## Abstract

Graft-versus-host disease (GVHD) remains the major cause of mortality and morbidity in non-relapse patients after allogeneic hematopoietic cell transplantation (allo-HCT). As the number of patients undergoing allo-HCT increases, it will become imperative to determine safe and effective treatment options for patients with GVHD, especially those who become refractory to systemic steroid therapy. Daratumumab (Dara), a humanized IgG1 (ĸ subclass) monoclonal antibody targeting the CD38 epitope, is used for the treatment of multiple myeloma. CD38 is a multifunctional ectoenzyme that behaves either as an enzyme, a cell adhesion molecule or a cell surface receptor involved in cell signaling. CD38 is also expressed on various immune effector and suppressor cells. However, the role of CD38 in the immune response remains elusive. We questioned whether CD38 is a potential therapeutic target against alloreactive T cells in the GVHD pathological process. Here, we investigated the impact of Dara on xenogeneic GVHD (xeno-GVHD) and graft-versus-leukemia (GVL) effects in a humanized murine model of transplantation, where human peripheral blood mononuclear cells were adoptively transplanted into immunocompromised NOD.SCID.gc-null (NSG) mice. Mice receiving Dara treatment experienced less weight loss, longer survival and lower GVHD scores compared with those in the control group. Histological evaluations, flow cytometry, RNA-sequencing and RT-qPCR analysis revealed that Dara efficaciously mitigated GVHD through multiple mechanisms including inhibition of the proliferation, activation and differentiation of CD8^+^ cytotoxic T cells, reduced expression of cytotoxic effector molecules, pro-inflammatory cytokines, chemokines and chemoattractant receptors by T cells and promotion of immunosuppressive T cells. More importantly, Dara preserved the GVL effect in a humanized mouse model of leukemia by metabolic reprograming of T cells to promote the induction of Th17, Th1/17and Tc1/17 cells. Our findings indicate that Dara may be an attractive therapeutic option to separate GVHD from GVL effects in patients with hematopoietic malignancies receiving allo-HCT.

## Introduction

Graft-versus-host disease (GVHD) remains the most common complication after allogeneic hematopoietic cell transplantation (allo-HCT), and is the major cause of mortality and morbidity in non-relapse patients. Despite the development of new therapies, 30%–50% of all allo-HCT patients develop acute GVHD (aGVHD) (1). GVHD is characterized by the attack of recipient host cells by allo-reactive donor T cells through the production of inflammatory cytokines, resulting in acute or chronic tissue damage. The current practice is to use broadly suppressive calcineurin inhibitors combined with methotrexate, sirolimus or mycophenolate mofetil to prevent GVHD. However, known off-target effects impair the beneficial graft-versus- leukemia (GVL) effects, which are also induced by the allogeneic T cell response (2). To date, corticosteroids remain the standard first-line treatment for aGVHD. While, in about 35%–50% of patients, aGVHD becomes refractory to systemic steroid therapy and is associated with high morbidity and mortality (3, 4). As the number of patients undergoing allo-HCT increases, it will become imperative to determine safe and effective treatment options for these patients, especially those who become refractory to systemic steroid therapy.

Daratumumab (Dara) is a humanized IgG1 (ĸ subclass) monoclonal antibody targeting the CD38 epitope, which has been approved by the USA Food and Drug Administration for the treatment of patients with relapsed and newly diagnosed multiple myeloma. Dara has a broad-spectrum killing ability against CD38-expressing tumor cells through multiple mechanisms including complement-dependent cytotoxicity, antibody-dependent cellular cytotoxicity, antibody-dependent cellular phagocytosis, and the induction of cell death through Fc-mediated crosslinking (5). CD38, a type II transmembrane glycoprotein, was first identified as an activation marker of T lymphocytes (6). CD38 is a multifunctional ectoenzyme that behaves either as an enzyme, a cell adhesion molecule or a cell surface receptor involved in cell signaling. CD38 is the main nicotinamide dinucleotide (NAD^+^) catabolic enzyme and metabolizes NAD to adenosine 59-diphosphate-ribose (ADPR) and cyclic ADP-ribose (cADPR), which result in the mobilization of calcium (Ca^2+^) (7). Intracellular Ca^2+^ participates in a variety of reactions such as cell proliferation, cell differentiation, and the activation and proliferation of lymphocytes.

However, the role of CD38 in the immune response remains elusive. Another important role for CD38 is the regulation of extracellular adenosine, which requires consumption of NAD^+^. Adenosine is important in immune modulation, being implicated in immune suppression through purinergic receptor binding and immunomodulation of multiple myeloma and lung cancer (7, 8). Because of the involvement of CD38 in the regulation of NAD^+^ and adenosine homeostasis, it is speculated that CD38 may function as an immune checkpoint molecule (9, 10). CD38 is expressed on the cells of several lineages, including B and T lymphocytes, and macrophages (11), and has been reported to be involved in neutrophil- and T cell-mediated immune responses (12, 13). Furthermore, CD38 promotes metabolic collapse in pathogens by degrading NAD^+^ and its precursors, and plays a role in the immune response to microbes (7). The inhibition of CD38 has therefore been proposed as an emerging therapeutic target in many autoimmune diseases, such as rheumatoid arthritis, systematic lupus erythematosus (7), asthma (14), neurodegeneration (15) and inflammatory bowel disease (16). Given its immunologically relevant functions, one published study evaluated the incidence of GVHD in 34 relapsed multiple myeloma patients treated with Dara after allo-HCT, in which the incidence of GVHD after Dara treatment was low, with only five patients (15%) developing aGVHD and no patient experiencing cGVHD (17). We questioned whether CD38 is a potential therapeutic target against alloreactive T cells in the GVHD pathological process. Here, we assessed the impact of Dara on xenogeneic GVHD (xeno-GVHD) and on the GVL effects in a humanized murine model of transplantation (human PBMC-infused NSG mice). We demonstrated that Dara efficaciously mitigated GVHD through multiple mechanisms including inhibition of the proliferation, activation and differentiation of CD8^+^ cytotoxic T cells, reduced expression of cytotoxic effector molecules, pro-inflammatory cytokines, chemokines and chemoattractant receptors by T cells, and promotion of immunosuppressive T cells. Importantly, Dara separated the GVL effects from GVHD by the metabolic reprograming of T cells to promote the induction of Th17, Th1/17and Tc1/17 cells.

## Materials and Methods

### Human Peripheral Blood Mononuclear Cell Collection

This study was approved by the Ethics Review Committee of Sir Run Run Shaw Hospital of Zhejiang University School of Medicine (Hangzhou, Zhejiang, China). Written informed consent was obtained from all participants in accordance with the Declaration of Helsinki. Peripheral blood samples were collected from healthy volunteers. Human peripheral blood mononuclear cells (hPBMCs) were separated from peripheral blood by Ficoll-Paque-Plus (GE Healthcare, Freiburg, Germany) gradient centrifugation.

### Human T-Cell Culture *In Vitro*

Human T cells were isolated using human T cell isolation kit (STEMCELL technologies, Vancouver, Canada). For T cell activation, human T cells were cultured in RPMI1640 with 10% FBS in T25 culture bottle (8×10^5^ cells per ml), stimulated with Dynabeads™ Human T-Expander CD3/CD28 (Thermo Fisher Scientific, Waltham, MA, USA) and incubated with 200 U/ml of recombinant human IL2 (Peprotech, Rocky Hill, NJ, USA) for 3 days. Then the dynabeads were removed and T cells were cultured in the presence of 50 μg/ml of Dara or human IgG control antibody for 48h. Next, human T cells were collected for flow cytometry analysis or metabolic detection.

### T-Cell Intracellular Metabolic Analysis

Human T cells (5×10^6^) were harvested and washed 3 times in cold PBS, and the cells were re-suspended by adding 0.5 ml stationary liquid containing 0.2ml methanol, 0.2ml acetonitrile and 0.1 ml water, quenched in liquid nitrogen for 10 minutes. Then, the samples were stored in the -80℃ refrigerator.

The sample was taken out from the -80°C refrigerator and thawed on ice. Then 500 μL 80% methanol-water internal standard extractant was added to the sample and vortex blended for 2 minutes. The mixture was frozen in liquid nitrogen for 5 minutes, placed on dry ice for 5 minutes, and thawed on ice for 5 minutes, finally vortex blended for 2 minutes, the whole process being circulated 3 times. Then the sample was centrifuged (12000 rpm, 4°C) for 10 minutes, 300 μl of the supernatant were transferred and standed still at -20°C for 30 minutes. The supernatant was centrifuged (12000 rpm, 4°C) again for 3 minutes and the final supernatant was used for metabolic analysis.

Intracellular metabolites were analyzed using Ultra-Performance Liquid Chromatography Mass Spectrometry (UPLC/MS) system at Metware Biotechnology (Wuhan, China). Mass spectrometric data were processed with Analyst 1.6.3 software (AB Sciex). Qualitative analysis was performed with built-in Metware database (MWDB) and the public database of metabolite information. Significantly changed metabolites between different groups were determined by VIP ≥1 and absolute Log_2_FC (fold change) ≥ 1. VIP values were extracted from OPLS-DA results, which also contain score plots and permutation plots, and generated using R package MetaboAnalystR. The data were log transform (log2) and mean centering before OPLS-DA. In order to avoid overfitting, a permutation test (200 permutations) was performed. Identified metabolites were annotated using KEGG Compound database, annotated metabolites were then mapped to KEGG Pathway database. Significantly enriched pathways were identified with a hypergeometric test’s p-value for a given list of metabolites.

### Xenogeneic Model of Graft-Versus-Host Disease

All animal studies were carried out following ethical approval from the Ethics Review Committee of Sir Run Run Shaw Hospital, Zhejiang University School of Medicine. NOD-Prkdc^scid^/IL2rg^tm1^/Bcgen (B-NSG/B-NDG) mice were purchased from the Shanghai Model Organism Center (Shanghai, China). All mice were bred and housed in a specific-pathogen-free (SPF) facility in microisolator cages and used at 8 to 10 weeks of age.

NSG mice were sublethally irradiated with 1.8 Gy total body irradiation on day ˗1, and given an intravenous injection of 1×10^7^ hPBMCs through the caudal vein on day 0. After hPBMC transplantation, mice were assessed for survival, weight and GVHD score every other day until the end of the experiment (day +50 post-transplantation). Engraftment of human white blood cells was detected in peripheral blood samples of mice 7, 14 and 21 days after transplantation. The severity score of GVHD was based on a scoring system that incorporates five clinical parameters: weight loss, posture, activity, skin integrity and fur ruffing (18). A severity scale of 0 to 2 was used for each parameter, with a maximum score of 10. Dara (100 mg/5 ml, Cilag AG, Schaffhausen, Switzerland) was solubilised in PBS at a final concentration of 0.5 μg/μl. To evaluate the effects of Dara on GVHD development, mice in the experimental group were intraperitoneally injected with Dara (5 mg/kg) once a week from day 0 to two weeks post-transplantation. In the control group, mice were intraperitoneally injected with human IgG control antibody (R&D Systems, Minneapolis, MN, USA).

Mice were ethically euthanized when they reached an aGVHD score of 6 or lost 25% of their body weight, or at 50 days post-transplantation.

Each experiment was performed using hPBMCs from a single donor. Each experimental group included six to eight mice, and the experiment was repeated three times (with three different donors to avoid inter-donor variability).

### Phenotypic Analysis by Flow Cytometry

Mouse spleen samples were mechanically dissociated. Splenocytes were filtrated into single cell suspensions by straining the cell suspension through a 70 μm cell strainer, then washing with fluorescence-activated cell sorting (FACS) buffer (1×DPBS plus 2% FBS). Next, splenocyte samples were incubated with human and mouse Fc receptor binding inhibitor (Fc Block, Biolegend, San Diego, CA, USA) at 4°C for 15 minutes to block Fc receptor binding, and then stained with the following antibodies specific for human antigens: fixable viability stain 510 (FVS510, BD Biosciences), anti-CD45-BV786 (BD Biosciences, clone HI30), anti-CD3-FITC/BB700 (BD Biosciences, clone HIT3a), anti-CD4-PE-CY7/BB700 (BD Biosciences, clone SK3), anti-CD8-APC-CY7/APC (BD Biosciences, clone SK1), anti-CD69-PE (Biolegend, clone FN50), anti-CD45RA-APC (Biolegend, clone HI100), anti-CCR7-BV421 (BD Biosciences, clone 3D12), anti-CD25-BV421/APC (BD Biosciences, clone M-A251) and anti-CXCR5-BB515 (BD Biosciences, clone RF8B2). Cells were incubated with antibodies against cell surface antigens at 4°C for 20 minutes in the dark, then washed with FACS buffer.

Intracellular antigen staining for Foxp3 and Ki67 was performed using the Foxp3/Transcription Factor Staining Buffer Kit (eBioscience) and antibodies including anti-Foxp3-PE (BD Biosciences, clone 259D/C7) and anti-Ki67-BV421 (BD Biosciences, clone B56). For intracellular cytokine staining, freshly harvested splenocytes were stimulated for 6 hours with Leukocyte Activation Cocktail (BD GolgiPlug™) prior to staining. Cells were then fixed and permeabilized using the Fixation/Permeabilization Solution Kit (BD Biosciences) and stained with antibodies including anti-IFNγ-APC (BD Biosciences, clone B27), anti-IL17A-PE (BD Biosciences, clone SCPL1362), anti-Granzyme A-PE (eBioscience, clone CB9) and anti-Granzyme B-PE (BD Biosciences, clone GB11).

Flow cytometry was performed using a Cytoflex LX flow analyzer (Beckman). Flow cytometry analyses were performed using the FlowJo software (Version 10.4).

### Histological Assay

Typical GVHD target organs (liver and lungs) obtained from mice in the xenogeneic GVHD model (19, 20) that had been euthanized at day 14 after transplantation were harvested, washed with PBS, fixed in 4% formaldehyde and embedded in paraffin. Five-μm sections were stained with hematoxylin-eosin (H&E) for histopathological analysis. Slides were coded without reference to prior treatment and examined in a blinded fashion for quantification. For immunohistochemical analysis, sections were stained with anti-human CD45 (CST, clone DM98I), anti-human CD4 (CST, clone EP204) and anti-human CD8 (CST, clone C8/144B). Slides were assessed using a semi-quantitative scoring system (grades 0 to 4.0) for abnormalities known to be associated with GVHD, as previously reported (21). Tissue damage in the lungs was scored according to the severity of inflammation and leukocyte infiltration (0 indicates no infiltration; 1, sporadic or <5% infiltration; 2, mild infiltration of 5%–25%; 3, moderate infiltration of 25%–50%; 4, severe infiltration of >60%). Four parameters were scored for the liver (portal infiltrate, biliary damage, centrilobular vein endotheliitis, apoptosis**)** and 0 indicated absent; 1, minimal; 2, mild and diffuse; 3, moderate; 4, severe (21).

Confocal images were acquired on an Olympus FV1200 Confocal Laser Scanning Microscope.

### Quantitative Real-Time PCR

Total RNA was extracted from cell samples using TRIzol reagent (Invitrogen, Carlsbad, CA, USA), and cDNA was transcribed with a reverse transcription kit (Vazyme, Nanjing, China) in accordance with the manufacturer’s protocols. Quantitative PCR (qPCR) assays were performed using ChamQ Universal SYBR qPCR Master Mix (Vazyme). Reactions were run on a LightCycler 480 real-time PCR system (Roche Diagnostics, Basel, Switzerland). The sequences of primers used for qRT-PCR are listed in **Table 1**.

**Table 1:** Quantitative real-time PCR primer sequences.

Gene	Forward primer (5’→3’)	Reverse primer (5’→3’)
*GAPDH*	GCACCGTCAAGGCTGAGAAC	TGGTGAAGACGCCAGTGGA
*TNF*	CCTCTCTCTAATCAGCCCTCTG	GAGGACCTGGGAGTAGATGAG
*IFNG*	AATTGTCTCCTTTTACTTCA	GTCATCTCGTTTCTTTTTGT
*IL17A*	TACAACCGATCCACCTCACC	CATGTGGTAGTCCACGTTCC
*GZMA*	AAAGACTGGGTGTTGACTGC	CCCTGGTTATTGAGTGAGCC
*GZMB*	TCCTGAGAAGATGCAACCAA	CCAGATCATAAGATAAGCCAT
*PRF1*	ACTTTGCAGCCCAGAAGACC	GTGCCGTAGTTGGAGATAAGC
*GNLY*	GGCTCCCTGCCCATAAAACA	GGGCTCTTGCCAGGTCGTAG
*TBX21*	ATGTGACCCAGATGATTGTGC	AAAGATATGCGTGTTGGAAGC
*CCR1*	CAAAGTCCCTTGGAACCAGA	GAGTTGCATCCCCATAGTCA
*CCR2*	CCACATCTCGTTCTCGGTTTATC	CAGGGAGCACCGTAATCATAATC
*CCR5*	TTGCCAAACGCTTCTGCAAAT	AGTGGATCGGGTGTAAACTGA
*CCL2*	AAACTGAAGCTCGCACTCTCG	TTGATTGCATCTGGCTGAGCG
*CCL3*	GGCTCTCTGCAACCAGTTCTC	TCGCTTGGTTAGGAAGATGACAC
*CCL4*	AAGCTCTGCGTGACTGTCCT	GCTGCTGGTCTCATAGTAATC
*CCL5*	CCCTCGCTGTCATCCTCATT	AGCACTTGCCACTGGTGTAG

### Serum Cytokine Levels

The sera of NSG mice were collected on day 14 after transplantation and the concentrations of human cytokines (IL6, IL10, IL17A, IFNγ, TNF) were determined using the Cytometric Bead Array Human Th1/Th2/Th17 Cytokine Kit (BD Biosciences).

### Human T Cell Isolation and RNA Sequencing Analysis

Viable human CD3^+^ T cells (FVS510^˗^ human CD45^+^ mouse CD45^˗^ human CD3^+^) were isolated from mouse splenocytes on day 14 post-transplantation using fluorescence-activated cell sorting (FACS) analysis (FACS Aria III, BD Biosciences) with antibodies, including anti-fixable viability stain 510 (FVS510, BD Biosciences), anti-human-CD45-PE (BD Biosciences, clone HI30), anti-mouse-CD45-FITC (BD Biosciences, clone 30-F11) and anti-human-CD3-percp/cy5.5 (BD Biosciences, clone HIT3a) according to the manufacturer’s instructions. The target number of sorted cells was one million.

Total RNA was extracted from sorted human CD3^+^ T cells using TRIzol reagent (Invitrogen) according to the manufacturer’s instructions. Subsequently, total RNA was qualified and quantified using a Nano Drop and Agilent 2100 Bioanalyzer (Thermo Fisher Scientific, Waltham, MA, USA). The mRNA was purified using oligo (dT)-attached magnetic beads for mRNA library construction, and purified mRNA was fragmented into small pieces with fragment buffer at the appropriate temperature. First-strand cDNA was generated using random hexamer-primed reverse transcription, followed by second-strand cDNA synthesis. Then, A-Tailing Mix and RNA Index Adapters were added to repair the ends. The cDNA fragments obtained from the previous step were acquired and amplified by PCR, and the products were purified using Ampure XP Beads and dissolved in EB solution. For quality control, the products were verified on the Agilent Technologies 2100 Bioanalyzer. The double-stranded PCR products from the previous step were heated denatured and circularized by the splint oligo sequence to obtain the final library. The single-stranded circular DNA was formatted as the final library. The final library was amplified with phi29 to obtain a DNA nanoball, which comprised more than 300 copies of one molecule, then DNA nanoballs were loaded into the patterned nanoarray and the BGIseq500 platform (BGI-Shenzhen, China) was used to generate single-end 50-base reads.

Fragments per kilobase of exon per million fragments mapped (FPKM) values were calculated based on the fragments’ length and read counts mapped to each fragment. The FPKMs for coding genes in six samples were calculated using Cuffdiff (v.1.3.0). Transcriptome FPKMs were the sum of the FPKMs of transcripts in each gene group. Significantly different expression levels in the digital transcript or gene expression datasets were determined using a model based on a negative binomial distribution with Cuffdiff (v.2.2.1). Comparisons with q-values <0.05 and absolute log2 (fold change) values ≥1 were considered as significantly differentially expressed.

### Xenogeneic Graft-Versus-Leukemia Models and Bioluminescence Imaging

We also evaluated whether Dara impacted on the GVL effect when it acted as an immunomodulatory factor. Nalm6 cells were engineered to express the green fluorescent protein (GFP) and luciferase reporter genes (Nalm6.LucGFP cells) for longitudinal tumor load monitoring by bioluminescence imaging. Female NSG mice at 8–12 weeks of age were irradiated (1.8 Gy) at day ˗1 and received intravenous injection of 1×10^5^ Nalm6.LucGFP cells or 1× 10^5^ Nalm6.LucGFP cells plus 1×10^7^ hPBMCs through the tail vein on day 0. Dara (5 mg/kg) or human IgG control antibody were intraperitoneally injected once a week for two weeks post-transplantation. Each experimental group included ten mice. Mice were assessed for survival every other day. Leukemia cell growth was evaluated weekly with bioluminescence imaging using the IVIS^®^ Lumina LT *in vivo* Imaging System (Perkin-Elmer). Imaging data were analyzed and quantified with Living Image 3.0 Software (Calipers).

### Statistical Analysis

Data were analyzed for statistical differences using Prism version 8.0 (GraphPad, San Diego, CA, USA). Flow cytometry data, RT-qPCR data, GVHD scores and serum cytokine levels were compared between the different groups using the Mann–Whitney test or ANOVA test. Differences in mouse survival rates were analyzed by the log-rank test. All probability values were generated from two-sided tests. *P < 0.05; **P < 0.01 and ***P < 0.001.

## Results

### Dara Significantly Alleviated Xeno-GVHD

**Figure 1:**
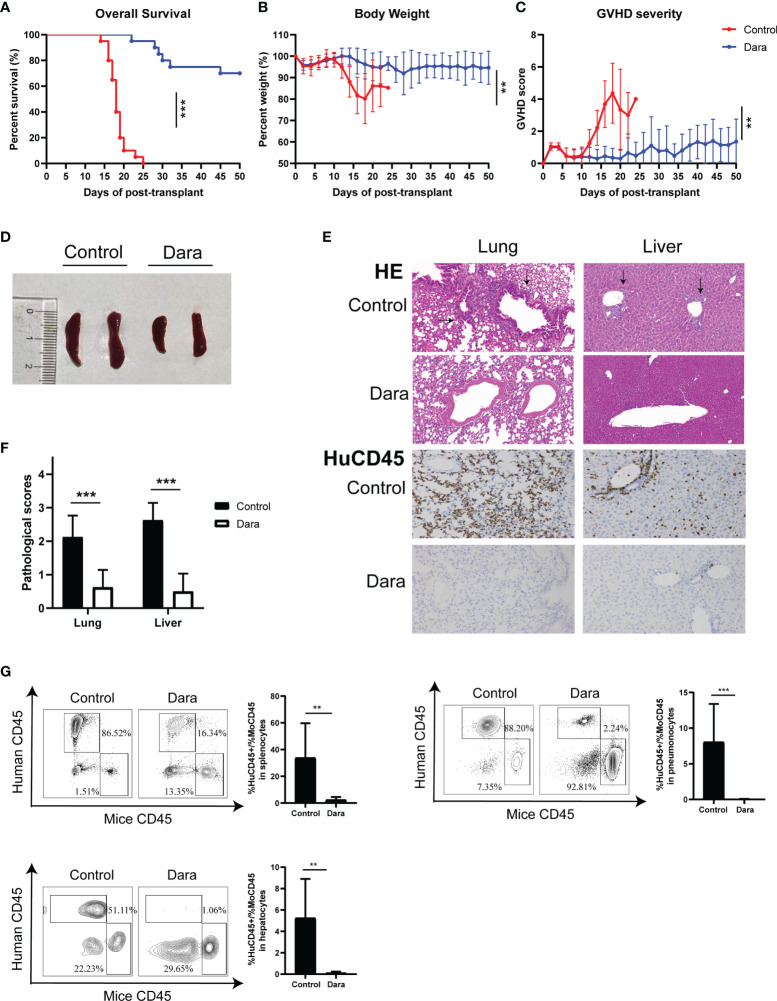
Dara alleviated xeno-GVHD. Mice receiving Dara treatment (n=20) achieved significantly longer survival **(A)**, less weight loss **(B)** and lower GVHD scores **(C)** compared with control mice (n=20). (D, E) Compared with mice receiving Dara treatment, mice in the control group had significantly enlarged spleens **(D)**, more evident tissue damage with lymphoid infiltration, endotheliitis, parenchymal alterations, hepatocyte apoptosis and portal tract expansion in the lung and liver, as determined by HE staining (original magnification, ×200), and less infiltration of human leukocytes, as determined by anti-human CD45^+^ (HuCD45^+^) staining in these GVHD targeted organs (original magnification, ×200) **(E)**. **(F)** Histopathological scores were reduced in the lung and liver of Dara-treated mice (n=8) compared with controls (n=8). **(G)** Flow cytometry analysis showed that the rates of engrafted human leukocytes (HuCD45^+^)/mouse leukocytes (MoCD45^+^) were significantly lower in splenocytes, and the lung and liver from mice in the Dara-treated group (n=8) than those in the control group (n=8) on day 14 post-transplantation. **P < 0.01 and ***P < 0.001.

To evaluate the impact of Dara on GVHD, NSG mice were sublethally irradiated on day ˗1 and given an intravenous injection of 1×10^7^ hPBMCs on day 0. Then, the mice were randomly assigned to groups either to be administered with Dara (5 mg/kg) or with human IgG control antibody once a week for 2 weeks post-transplantation. As shown in **Figure 1A**, survival was significantly longer in mice receiving Dara compared with control mice. The median survival time of mice in the control group was 18 days, whereas 70% of mice receiving Dara treatment survived from GVHD for more than 50 days (P < 0.001). Consistent with this survival advantage, mice receiving Dara treatment experienced less weight loss (**Figure 1B**) and lower GVHD scores (**Figure 1C**) compared with those in the control group. Histological evaluations were performed on mice euthanized on day 14 post-transplantation. Compared with mice receiving Dara treatment, mice in the control group had significantly enlarged spleens (**Figure 1D**). As shown in **Figure 1E**, HE staining of the lung and liver tissue obtained from IgG isotype control-treated mice demonstrated evident tissue damage with lymphoid infiltration, endotheliitis, parenchymal alterations, hepatocyte apoptosis and portal tract expansion. In Dara-treated mice, staining of the lung and liver tissue revealed only focal infiltrates and overall subnormal histology. Furthermore, anti-human CD45^+^ (HuCD45^+^) staining in these GVHD-targeted organs confirmed strikingly less abundant infiltrates of human leukocytes in the organs obtained from Dara-treated mice (**Figure 1E**). Upon blinded evaluation, we obtained drastically reduced histopathological scores for the lung and liver (median lung score: 0.63 ± 0.52 vs 2.13 ± 0.64, P < 0.001; median liver score: 0.5 ± 0.53 vs 2.63 ± 0.52, P < 0.001) of Dara-treated mice compared with control mice (**Figure 1F**). Flow cytometry analysis showed that the rate of engrafted human leukocytes (HuCD45^+^)/mouse leukocytes (MoCD45^+^) was 2.53 ± 2.03 in splenocytes on day 14 post-transplantation in the Dara-treated group, which was significantly lower than that in the control group (33.88 ± 25.83, P = 0.0041). The same trend was observed in GVHD-targeted organs, i.e., the lung (0.03 ± 0.04 *vs* 8.06 ± 5.33, P < 0.001) and liver (0.11 ± 0.12 *vs* 5.25 ± 3.65, P = 0.0014) (**Figure 1G**).

### Dara Reduced Human T Cell Infiltration in GVHD-Targeted Organs and Induced Skewed Proportions of T Cell Functional Subsets

**Figure 2:**
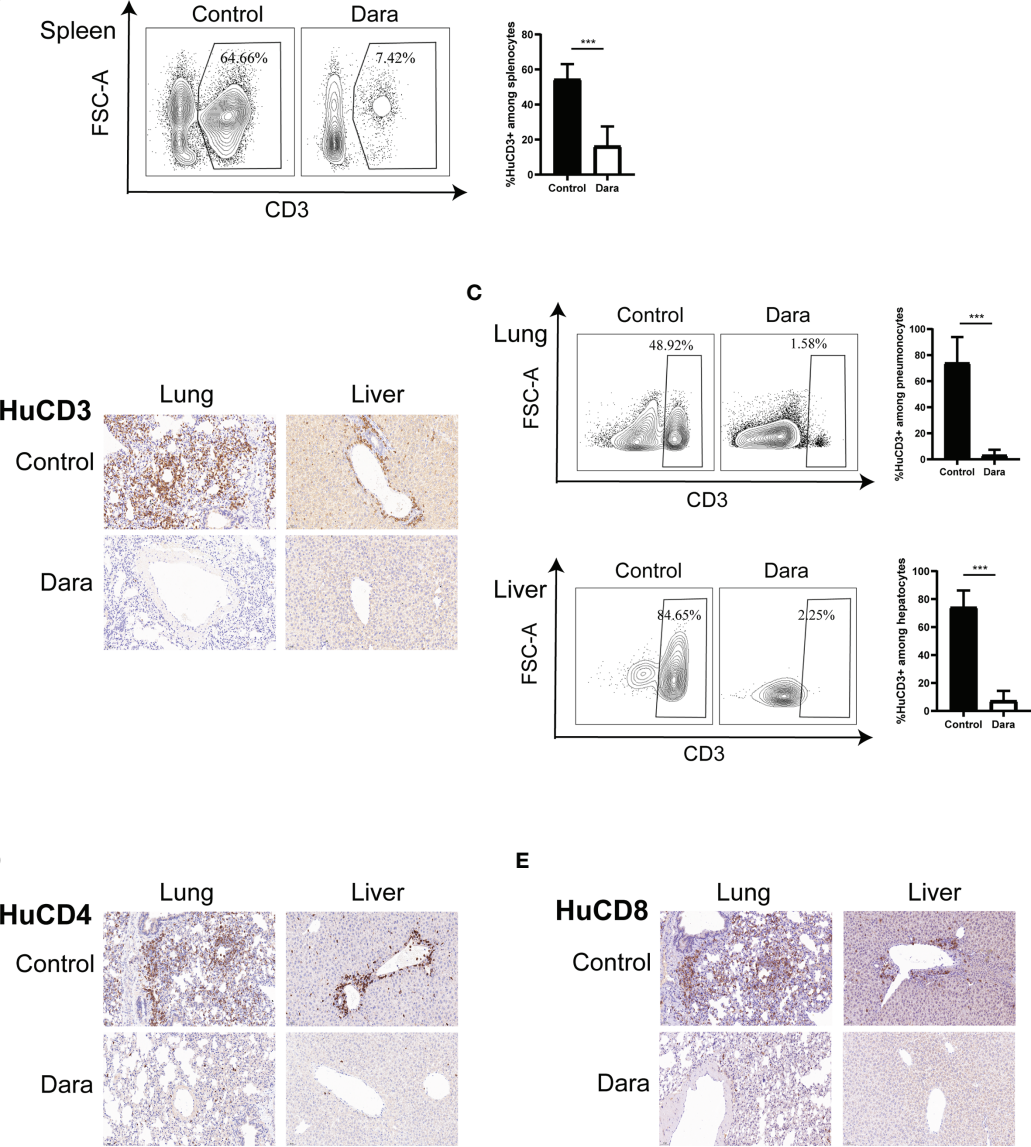
Dara reduced human T cell infiltration in GVHD-targeted organs. **(A)** The proportion of human CD3^+^ T cells engrafted in splenocytes was reduced in mice in the Dara-treated group (n=8) compared with those in the control group (n=8) on day 14 post-transplantation. **(B–E)** Flow cytometry and immunohistochemical staining (original magnification, ×200) showed that the infiltration of human CD3^+^, CD4^+^ and CD8^+^ T cells in GVHD-targeted organs (lung and liver) was significantly reduced in mice treated with Dara compared with control mice. ***P < 0.001.

In light of the infiltration differences of HuCD45^+^ cells between control and Dara-treated mice on day 14 post-transplantation, we examined whether the proportion of functional subsets and activation status of human T cells were affected by Dara. The proportion of engrafted human CD3^+^ T cells in splenocytes in Dara-treated mice was significantly reduced compared with those in control mice (Dara: 16.29% ± 11.21% *vs* Control: 54.45% ± 8.56%, P < 0.0001) (**Figure 2A**). Furthermore, flow cytometry and immunohistochemical staining showed that the infiltration of human CD3^+^ (**Figures 2B, C**), CD4^+^ (**Figure 2D**) and CD8^+^ (**Figure 2E**) T cells in GVHD-targeted organs (lung and liver) were significantly reduced in mice treated with Dara compared with control mice. The proportions of infiltrated human CD3^+^ T cells in lung and live in Dara-treated mice were 3.23% ± 4.18% and 7.31% ± 7.03%, which were 73.95% ± 19.92% (P < 0.0001) and 74.23% ± 11.99% (P < 0.0001) in control mice, respectively (**Figure 2C**).

**Figure 3:**
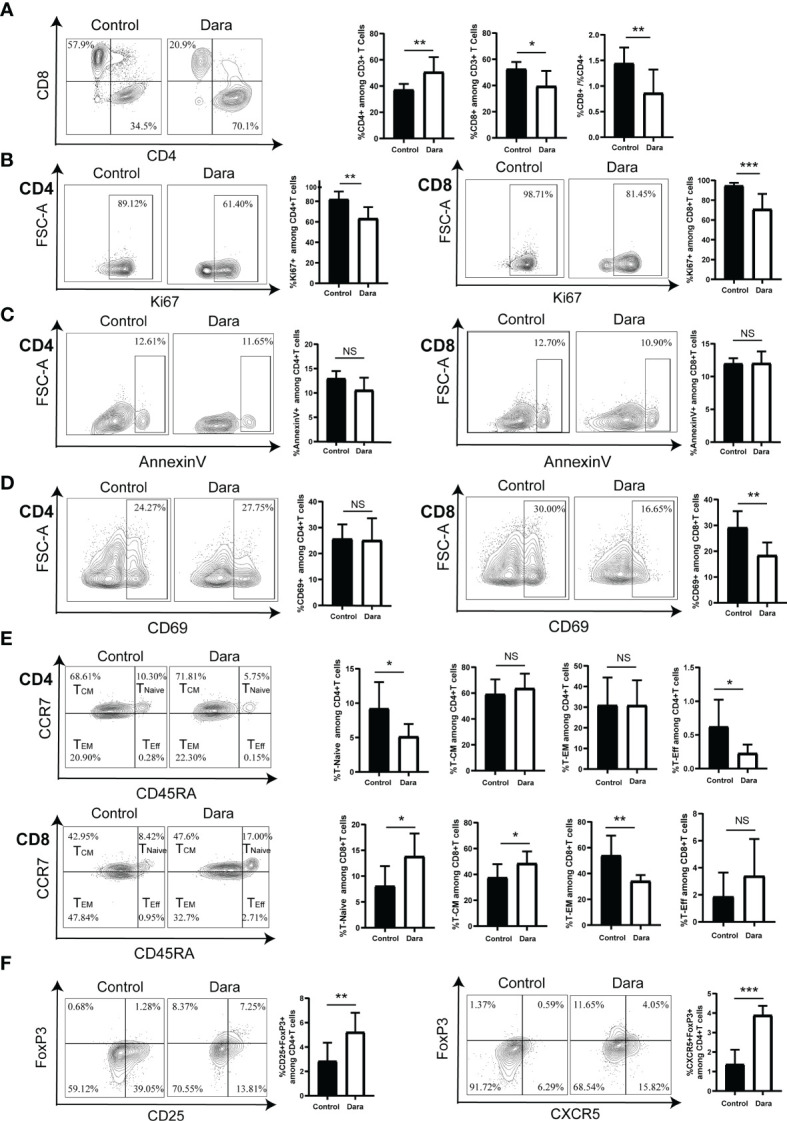
Dara induced a skewed proportion of T cell functional subsets. **(A)** A reduced frequency of CD8^+^ T cells, a higher frequency of CD4^+^ T cells, as well as a lower ratio of CD8^+^/CD4^+^ T cells were observed in the splenocytes of Dara-treated mice (n=8) compared with those from control mice (n=8) on day 14 post-transplantation. **(B)** Dara decreased the expression levels of Ki67 both on CD4^+^ T cells and CD8^+^ T cells. **(C)** Dara had no significant effect of induction of apoptosis of T cells. The apoptosis rates in CD4^+^ and CD8^+^ T cells from Dara-treated mice were comparable to those in T cells from control mice. **(D)** A significantly decreased frequency of CD69^+^ cells among CD8^+^ T cells was observed in Dara-treated mice, while this trend was not observed among CD4^+^ T cells. **(E)** CD8^+^ T cell subpopulations in splenocytes from Dara-treated mice showed enhanced frequencies of naïve T cells (CD45RA^+^CCR7^+^) and central memory T cells (T_CM_, CD45RA^˗^CCR7^+^), a decreased frequency of effector memory T cells (T_EM,_ CD45RA^˗^CCR7^˗^) and no change in the frequency of terminally differentiated effector T cells. While, no inhibitory effect of Dara on CD4^+^ T cell differentiation was observed, with no significant increase in the frequencies of naïve/memory phenotypes. **(F)** Flow cytometry analysis showed that both the frequencies of Treg (CD4^+^CD25^+^FoxP3^+^) and Tfr (CD4^+^CXCR5^+^FoxP3^+^) cells were significantly higher in splenocytes obtained from Dara-treated mice than those from control mice at 14 days post-transplantation. *P < 0.05; **P < 0.01 and ***P < 0.001; NS P > 0.05.

To assess the impact of Dara on specific T cell functional subsets, we further evaluated the proportions of human CD4^+^ T cells and CD8^+^ T cells in human CD3^+^ T cells in splenocytes from Dara-treated mice and control mice on day 14 post-transplantation. We observed a reduced frequency of CD8^+^ T cells (Dara: 39.54% ± 11.62% *vs* Control: 52.6% ± 5.36%, P = 0.012), a higher frequency of CD4^+^ T cells (Dara: 50.76% ± 11.35% vs Control: 37.24% ± 4.41%, P = 0.0072) and a lower ratio of CD8^+^/CD4^+^ T cells in Dara-treated mice (Dara: 0.86 ± 0.46 *vs* Control: 1.44 ± 0.31, P = 0.01) (**Figure 3A**).

To investigate whether the decreased ratio of CD8^+^/CD4^+^ T cells in Dara-treated mice originates solely from the anti-proliferation effect of the drug on CD8^+^ T cells or whether there is also more significant inhibition on CD8^+^ T cell proliferation than CD4^+^ T cell proliferation, we compared the frequency of human Ki67^+^CD8^+^ T cells and Ki67^+^CD4^+^ T cells in control and Dara-treated mice. We found that Dara decreased the expression levels of Ki67 both on CD4^+^ T cells (Dara: 63.36% ± 11.04% vs Control: 82.16% ± 7.5%, P = 0.0014) and CD8^+^ T cells (Dara: 70.9% ± 15.43% *vs* Control: 94.65% ± 2.9%, P < 0.001), which suggested that Dara could inhibit both the proliferation of CD4^+^ and CD8^+^ T cells (**Figure 3B**). On the other hand, we evaluated whether the reduced frequency of CD8^+^ T cells in splenocytes may be contributed to Dara-mediated apoptosis of CD8^+^ T cells or not. The apoptosis rates were 10.58% ± 2.56% in CD4^+^ T cells and 12.03% ± 1.79% in CD8^+^ T cells from Dara-treated mice, which were comparable to those in T cells from control mice (12.97% ± 1.55% in CD4^+^ T cells, 11.98% ± 0.8% in CD8^+^ T cells, P >0.05). Dara had no significant effect of induction of apoptosis of T cells (**Figure 3C**). Finally, taking into account that CD38 is an activation marker of T lymphocytes, we next examined whether the activation status of human T cells was affected by Dara. Since CD69 is an established activation marker of T cells, we assessed the expression level of CD69 on T cells. A significantly decreased frequency of CD69^+^ cells in CD8^+^ T cells was observed in Dara-treated mice (Dara: 18.41% ± 4.98% *vs* Control: 29.23% ± 6.31%, P = 0.0019), whereas this trend was not observed in CD4^+^ T cells (Dara: 25.11% ± 8.47% *vs* Control: 25.65% ± 5.57%, P = 0.88) (**Figure 3D**). These data support the hypothesis that Dara mainly inhibits CD8^+^ T cell activation.

Next, we assessed the impact of Dara on human T cell differentiation from naive to memory/effector phenotypes. Human T cell differentiation markers (CD45RA/CCR7) were analyzed by flow cytometry. Human CD8^+^ T cell subpopulations in splenocytes from Dara-treated mice showed enhanced frequencies of naïve T cells (CD45RA^+^CCR7^+^) (Dara: 13.84% ± 4.42% vs Control: 8.08% ± 3.86%, P = 0.0148) and central memory T cells (T_CM_, CD45RA^˗^CCR7^+^) (Dara: 48.56% ± 9.26% vs Control: 37.58% ± 10.29%, P = 0.0415), a decreased frequency of effector memory T cells (T_EM,_ CD45RA^˗^CCR7^˗^) (Dara: 34.2% ± 4.65% vs Control: 53.99% ± 15.47%, P = 0.0038) and no change in the frequency of terminally differentiated effector T cells (T_Eff_, CD45RA^+^CCR7^˗^) (Dara: 3.39% ± 2.73% *vs* Control: 1.87% ± 1.77%, P = 0.21). Dara did not exert any inhibitory effect on CD4^+^ T cell differentiation, with no significant increase in the frequencies of naïve/memory phenotypes (**Figure 3E**).

Finally, we assessed the impact of Dara on regulatory T (Treg) cells. We detected the proportions of CD4^+^CD25^+^FoxP3^+^ T cells (Treg) and CD4^+^CXCR5^+^FoxP3^+^ T cells (follicular regulatory T cells, Tfr), which were identified as important immunosuppressive T cells in GVHD pathogenesis, in splenocytes on day 14 post-transplantation. Flow cytometry analysis showed that both the frequencies of Treg (Dara: 5.23% ± 1.58% vs Control: 2.84% ± 1.51%, P = 0.008) and Tfr (Dara: 3.89% ± 0.48% vs Control: 1.37% ± 0.75%, P < 0.001) were significantly higher in splenocytes obtained from Dara-treated mice than those from control mice (**Figure 3F**). Taken together, our data suggested that Dara may alleviate xeno-GVHD not only by inhibition of the proliferation, activation and differentiation of CD8^+^ cytotoxic T cells, but also by increasing the frequency of immunosuppressive T cells.

To confirm the effects of Dara on T cell functions assayed in the xeno-GVHD animal model, we further performed experiments to assay the effects of Dara on T cell apoptosis, proliferation, activation, differentiation as well as Treg induction *in vitro*. Human T cells were cultured in the presence or absence of Dara at 50ug/ml for 48h. Dara had no significant effect of induction of apoptosis on T cells, in which the apoptosis rates were only around 1-2% after being cultured with Dara (**Supplementary Figure S1**). There is also no significant inhibition either on CD4^+^ T cell proliferation or on CD8^+^ T cell proliferation. We found that the expression levels of Ki67 on CD4^+^ T cells and CD8^+^ T cells were comparable in Dara-treated T cells and human IgG control-treated T cells (**Supplementary Figure S2**). A significantly decreased frequencies of CD69^+^ cells in both CD4^+^ T cells and CD8^+^ T cells were observed in Dara-treated T cells (**Supplementary Figure S3**). These data were consistent with the results assayed in the xeno-GVHD animal model that Dara inhibits T cell activation. Although Dara did not exert any inhibitory effect either on CD4^+^ T cell differentiation or on CD8^+^ T cell differentiation *in vitro* culture, with no significant increase in the frequencies of naïve/memory phenotypes (**Supplementary Figure S4**). Consistent with the results assayed in the xeno-GVHD animal model, flow cytometry analysis showed that the frequency of Treg was significantly higher in Dara-treated T cells than those from human IgG control-treated T cells (**Supplementary Figure S5**).

### The Impact of Dara on the Whole Gene Expression Profile in Human T Cells Involved in Xeno-GVHD and the Effect of Dara on the Metabolic Regulation of T Cells

**Figure 4:**
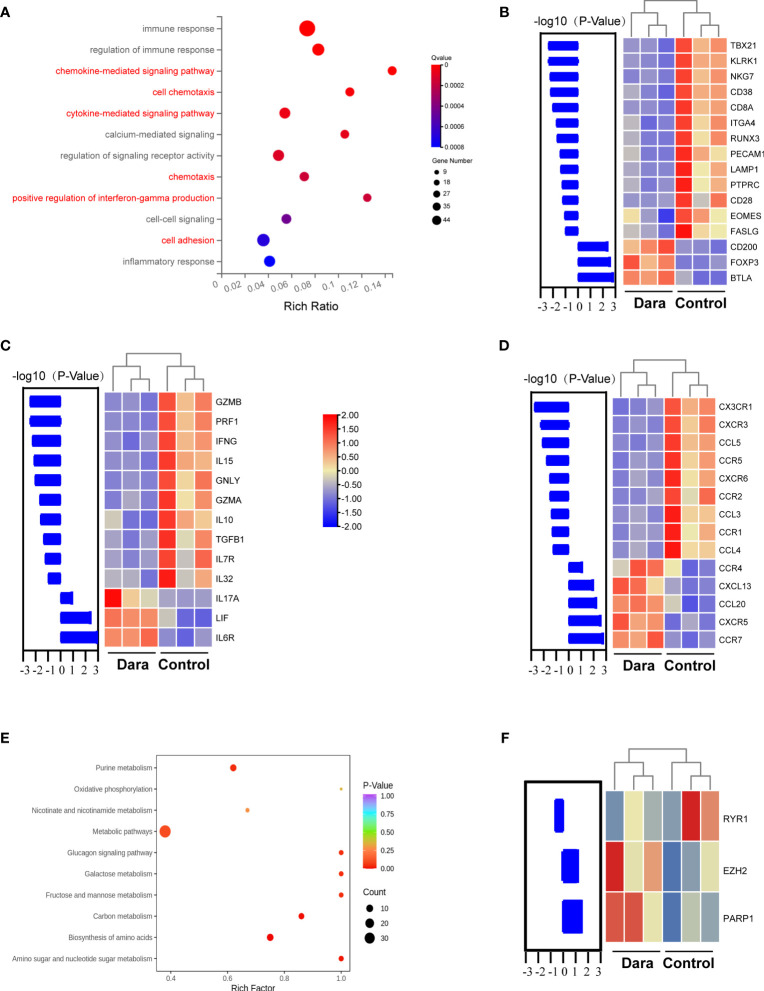
The impact of Dara on the whole gene expression profile of human T cells involved in xeno-GVHD and the effect of Dara on the metabolic regulation of T cells. **(A)** Transcriptome sequencing of sorted human CD3^+^ T cells engrafted into the spleen obtained from Dara-treated mice (n=3) or those from control mice (n=3) at 14 days post-transplantation. Pathway analysis of genes regulated by Dara in T cells. **(B)** Significantly decreased expression of transcription factors involved in the induction of T cell cytotoxic reactions and type I T cell differentiation, activation/immune response markers and adhesion molecules, was observed in human T cells from Dara-treated mice compared with those from control mice. While, significantly increased expression of transcription factor of Treg and Tfr, the FoxP3 gene, and genes known to suppress the immune response, were observed in human T cells from Dara-treated mice. **(C, D) ** Genotypic characterization of infiltrated human T cells in Dara-treated mice revealed decreased expression of pro-inflammatory cytokines, cytotoxic effector molecules, chemokines and chemoattractant receptors. **(E)** Intracellular metabolic changes in human T cells after being cultured in the presence of Dara at 50ug/ml for 48h. **(F)** Genotypic characterization of infiltrated human T cells in Dara-treated mice revealed changed transcription levels of RYR1, PARP1, EZH2.

To further explore the mechanisms by which Dara inhibits T cell activity, we performed transcriptome sequencing of sorted human CD3^+^ T cells engrafted into the spleens obtained from Dara-treated mice or those from control mice on day 14 post-transplantation. Data analysis revealed 358 genes that were significantly differentially expressed (log2 Ratio| ≥ 1, *q* < 0.05), relative to the T cell controls, in human CD3^+^ T cells receiving Dara-treatment. Pathway analysis revealed a remarkable abundance of gene signatures involved in pathways of the immune response, chemokine-mediated signaling pathway, cell chemotaxis, cytokine-mediated signaling pathway, positive regulation of interferon-gamma (IFNγ) production and cell adhesion (**Figure 4A**). Further analysis identified T cell immune reaction as the process most altered at the gene expression level. Significantly decreased expression of transcription factors involved in the induction of the T cell cytotoxic reaction and type I T cell differentiation, such as TBX21/T-bet (T-box transcription factor 21), RUNX3 (RUNX family transcription factor 3) and EOMES (eomesodermin), were observed in human T cells from Dara-treated mice. Engrafted human T cells from Dara-treated mice also exhibited reduced expression of activation/immune response markers including KLRK1/NKG2D (killer cell lectin like receptor K1), NKG7 (natural killer cell granule protein 7), LAMP1/CD107a (lysosomal-associated membrane protein 1), CD28 and adhesion molecules including PECAM1/CD31 (platelet and endothelial cell adhesion molecule 1) and ITGA4/CD49d (integrin subunit alpha 4). Whereas, significantly increased expression of transcription factor of Treg and Tfr, the FoxP3 gene, and increased expression of CD200/OX-2 and CD272/BTLA (B and T lymphocyte associated), which are known to suppress the immune response, were observed in human T cells from Dara-treated mice (**Figure 4B**).

Further genotypic characterization of infiltrated human T cells in Dara-treated mice revealed decreased expression of cytotoxic effector molecules, cytokines, chemokines and chemoattractant receptors. Decreased expression of pro-inflammatory cytokine and cytotoxic effector molecule genes including IL15, IL10, IL32, IFNG, PRF1/perforin, GZMA/Granzyme A, GZMB/Granzyme B and GNLY/Granulysin were observed in Dara-treated T cells (**Figure 4C**). Furthermore, Dara-treated T cells also displayed decreased transcription levels of chemokines and chemoattractant receptors, which play pivotal roles in lymphocyte migration to secondary lymphoid organs or to peripheral tissues under T cell adaptive immune reactions, including CX3CR1, CXCR3, CCR1, CCR2, CCR5, CCL3, CCL4 and CCL5 (**Figure 4D**). Consistent with the high expression of transcription factors of Tfr cells, the CXCR5 chemokine receptor gene was increased in expression in T cells from Dara-treated mice. Intriguingly, increased expression levels of the type 17 T cell (T17)-related cytokine and chemokine, IL17A and CCL20, were observed in T cells from Dara-treated mice (**Figures 4C, D**).

Taking into account that the NAD^+^ glycohydrolase (NADase) activity of CD38 which determines the intracellular level of NAD^+^, a principal metabolite regulating diverse biochemical and cellular processes. We detected intracellular metabolic changes in T cells after human T cell being cultured in the presence of Dara at 50ug/ml or human IgG control for 48h. Data analysis revealed that Dara modulated metabolic pathways of T cells involving purine metabolism, oxidative phosphorylation, nicotinate and nicotinamide metabolism, *etc* (**Figure 4E**). More importantly, consistent with the results of Dara on the metabolic regulation of T cells *in vitro*, transcriptome sequencing data of sorted human CD3^+^ T cells engrafted into the spleens obtained from Dara-treated xeno-GVHD mice also displayed changed transcription levels of pivotal enzymes and/or epigenetic modifiers including ryanodine receptor 1 (RYR1), poly (ADP-ribose) polymerase (PARP), enhancer of zeste homolog 2 (EZH2) genes (**Figure 4F**), which were involved in CD38 mediated regulation of metabolic pathways and chromatin modifications in T cells (22).

### Dara Alleviated Xeno-GVHD by Inhibition of T Cell Activation and Migration

The results of RNA sequencing (RNA-seq) suggested that Dara mitigated xeno-GVHD by inhibition of T cell activation and migration. For further confirmation, we detected the abundance of T cell functional subsets including Granzyme A^+^, Granzyme B^+^, IFNγ^+^ and IL17A^+^ T cells by flow cytometry as well as the mRNA expression levels of pro-inflammatory cytokines, cytotoxic effector molecules, chemokines and chemoattractant receptors including the *IFNG*, IL17A, TBX21/T-bet, GZMA/Granzyme A, GZMB/Granzyme B, GNLY/Granulysin, PRF1/perforin, CCL2, CCL3, CCL4, CCL5, CCR1, CCR2 and CCR5 genes by RT-qPCR in human T cells engrafted in splenocytes from Dara-treated or control mice on 14 days post-transplantation. As shown in **Table 2**, the reduced proportions of human IFNγ^+^IL17A^˗^CD4^+^ T (Th1) and IFNγ^+^IL17A^˗^CD8^+^ T (Tc1) cells, the lower expression levels of IFNG mRNA among CD4^+^ and CD8^+^ T cells, as well as the diminished expression of TBX21/T-bet mRNA in human T cells in splenocytes were observed in Dara-treated mice. Furthermore, serum IFNγ (Dara: 132.25 ± 73.41pg/ml *vs* Control: 509.21 ± 248.4 pg/ml, P = 0.0001), IL6 (Dara: 1.15 ± 0.49 pg/ml *vs* Control: 2.47 ± 0.83 pg/ml, P = 0.0002) and IL10 (Dara: 1.92 ± 2.36 pg/ml vs Control: 24.06 ± 23.44 pg/ml, P = 0.0054) concentrations were also significantly lower in Dara-treated mice than in control mice. Granzyme A, Granzyme B, Granulysin and Perforin 1 secretion by T cells has been shown to play pivotal functions in T cell cytotoxicity. Dara treatment significantly reduced the frequencies of Granzyme A-secreting CD8^+^ T cells (Dara: 53.05% ± 9.07% vs Control: 70.05% ± 8.01%; P = 0.0011) and Granzyme B-secreting CD8^+^ T cells (Dara: 31.48% ± 9.97% vs Control: 68.21% ± 8.53%, P < 0.0001). Furthermore, mRNA expression levels of GZMA/Granzyme A, GZMB/Granzyme B, GNLY/Granulysin and PRF1/perforin genes in human engrafted CD8^+^ T cells were also decreased. These data support that Dara inhibits T cell activation of both effector Th1 and Tc1 cells. Interestingly, in agreement with the RNA-seq results, Dara may induce Th17 (IFNγ^˗^IL17A^+^CD4^+^), Th1/17 (IFNγ^+^ IL17A^+^ CD4^+^), Tc17 (IFNγ^˗^IL17A^+^CD8^+^) and Tc1/17 (IFNγ^+^IL17A^+^CD8^+^) proliferation, as well as *IL17* mRNA levels in splenocytes, and the serum IL17 concentration was increased (**Table 2**).

**Table 2:** Impact of Dara on engrafted human T cell activation and migration.

Parameter*	Method	Control (n=8)		Dara (n=8)		p-value
		Median	SD	Median	SD	
**Sacrifice at day14, spleen**						
Functional subsets **among CD4^+^ T cells (%)**	FACS^*^					
IFNγ^+^ IL17A^-^ T cells (Th1)		76.31	3.52	60.15	1.56	<0.0001
IFNγ^-^ IL17A^+^ T cells (Th17)		0.4	0.16	1.9	0.63	<0.0001
IFNγ^+^ IL17A^+^ T cells (Th1/17)		4.65	1.69	9.61	1.7	<0.0001
Functional subsets **among CD8^+^ T cells (%)**	FACS					
IFNγ^+^ IL17A^-^ T cells (Tc1)		95.13	0.62	84.34	4.11	<0.0001
IFNγ^-^ IL17A^+^ T cells (Tc17)		0.07	0.06	0.89	0.84	0.015
IFNγ^+^ IL17A^+^ T cells (Tc1/17)		1.84	0.66	4.41	1.33	0.0002
Granzyme A^+^ T cells		70.05	8.01	53.05	9.07	0.0011
Granzyme B^+^ T cells		68.21	8.53	31.48	9.97	<0.0001
**Serum cytokine at day14 (pg/ml)**	CBA^*^					
IFNγ		509.21	248.4	132.25	73.41	0.0001
IL6		2.47	0.83	1.15	0.49	0.0002
IL10		24.06	23.44	1.92	2.36	0.0054
IL17A		1.32	1.39	4.23	2.77	0.0054
**Sacrifice at day14, spleen**						
**Gene expression levels of T cells**	RT-qPCR					
**Pro-inflammatory cytokine gene expression among CD4^+^ -T cells**	RT-qPCR					
*IFNG*		0.83	0.16	0.47	0.18	0.0275
*IL17A*		0.72	0.48	8.93	6.03	0.0348
TBX21/T-bet		0.95	0.08	0.77	0.08	0.021
**Cytotoxic effector molecule gene expression among CD8^+^-T cells**	RT-qPCR					
*IFNG*		1.32	0.28	0.89	0.2	0.04
*IL17A*		0.76	0.49	2.71	0.88	0.0081
GZMA/Granzyme A		1.06	0.15	0.63	0.16	0.0069
GZMB/Granzyme B		1.15	0.26	0.55	0.28	0.0208
GNLY/Granulysin		1.17	0.2	0.23	0.11	0.0002
PRF1/perforin		1.06	0.06	0.6	0.3	0.0218
**CD4^+^-T cell related chemokine and chemoattractant receptor gene expression**	RT-qPCR					
* CCL2*		0.86	0.18	0.45	0.2	0.0229
* CCL3*		1.05	0.27	0.5	0.21	0.0176
* CCL5*		0.96	0.13	0.44	0.15	0.0022
* CCR1*		1.11	0.33	0.62	0.18	0.0387
* CCR2*		0.73	0.27	0.25	0.08	0.014
* CCR5*		0.92	0.13	0.58	0.25	0.0498
**CD8^+^-T cell related chemokine and chemoattractant receptor gene expression**	RT-qPCR					
* CCL3*		0.95	0.15	0.49	0.2	0.0098
* CCL4*		1.08	0.15	0.43	0.09	0.0003
* CCR1*		1.42	0.32	0.76	0.3	0.0239
* CCR2*		0.83	0.25	0.41	0.2	0.0413

*All parameters indicate human cells. Data show median values for 8–10 mice/condition with the interquartile range (IQR). FACS: flow cytometric analysis; CBA: Cytometric Bead Array.

Dara treatment also inhibited the mRNA expression of CD4^+^ T cell-related chemokine and chemoattractant receptor genes including CCL2, CCL3, CCL5, CCR1, CCR2 and CCR5, and CD8^+^ T cell-related chemokine and chemoattractant receptor genes including CCL3, CCL4, CCR1 and CCR2 (**Table 2**). Taken together, these data suggest that the protective effect against GVHD conferred by Dara treatment was highly associated with the immunomodulatory effects of both effector CD4^+^ and CD8^+^ T cell activation and migration.

### Dara Preserves the GVL Effect

The ideal treatment strategy to target GVHD would be to inhibit GVHD without dampening the GVL effect. To investigate whether the immunomodulatory function of Dara could affect GVL activity, we used a human leukemia cell line Nalm6 transfected to express luciferase (Nalm6.LucGFP). NSG mice were transplanted with 1×10^5^ Nalm6.LucGFP cells with or without 1×10^7^ hPBMCs cells. Mice were then treated with or without Dara. The elimination or growth of leukemia cells was evaluated using an *in vivo* bioluminescent imaging system.

**Figure 5:**
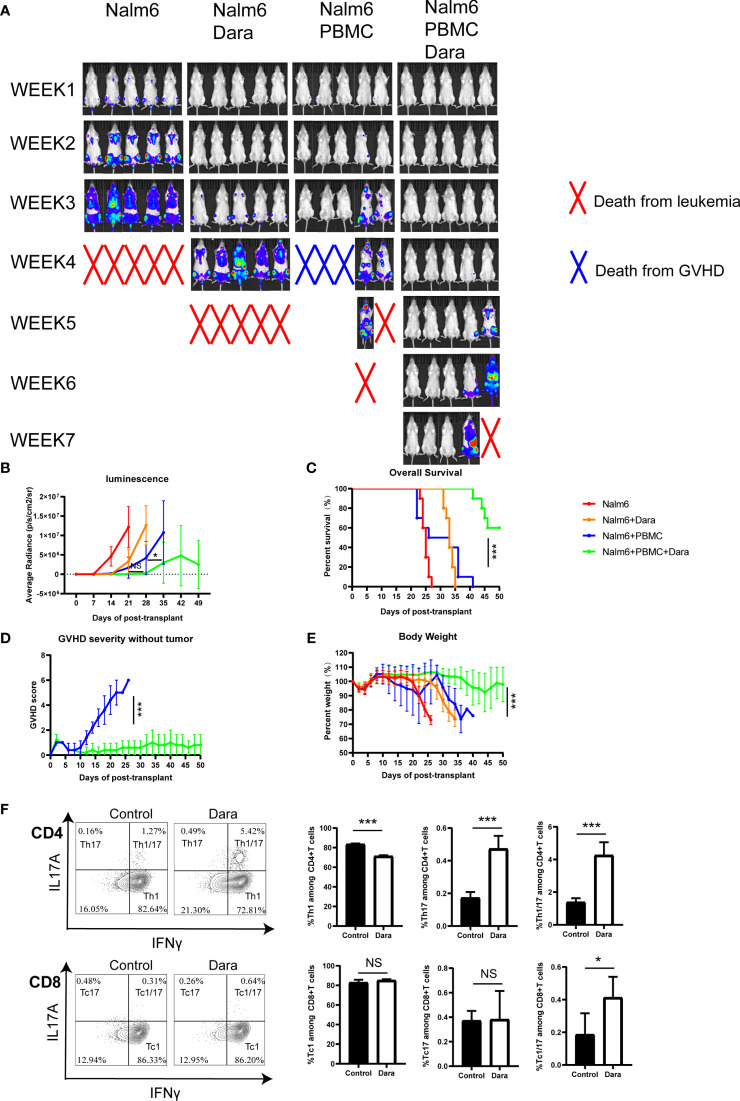
Dara preserves the GVL effect in a humanized mouse model of leukemia. Each experimental group included 10 mice. **(A, B)** Bioluminescence imaging (5 mice each experiment group presented) and quantification of bioluminescence (n=10) revealed that treatment with hPBMCs significantly reduced leukemia progression in the presence or absence of Dara during 3-4weeks post transplantation. After 4 weeks, this reduction in leukemia progression was most significant after treatment with hPBMCs in combination with Dara compared with hPBMCs treatment alone (P < 0.05). **(C)** Mice administered hPBMCs in combination with Dara treatment (n=10) showed superior survival rates compared with mice administered hPBMCs alone (n=10) or Dara treatment alone (n=10). **(D, E)** Compared with the high GVHD scores in mice receiving hPBMC alone, no GVHD **(D)** and no significant weight loss **(E)** were detected in mice receiving hPBMCs in combination with Dara. **(F)** Dara induced Th17 (IFNγ^˗^IL17A^+^CD4^+^), Th1/17 (IFNγ^+^ IL17A^+^CD4^+^) and Tc1/17 (IFNγ^+^IL17A^+^CD8^+^) proliferation in the humanized NSG-leukemia model. *P < 0.05; ***P < 0.001; NS P > 0.05.

Mice receiving Nalm6 cells without Dara or hPBMC died from leukemia. Interestingly, the treatment of Dara alone prolonged the survival of mice indicating the potential inhibition of Nalm6 proliferation by Dara, although a relapse of leukemia was observed in mice treated with Dara alone. Bioluminescence imaging and quantification of bioluminescence revealed that treatment with hPBMCs significantly reduced leukemia progression irrespective of the presence or absence of Dara during 3-4weeks post transplantation, although after 4 weeks, this reduction in leukemia progression was most significant after treatment with hPBMCs in combination with Dara compared with hPBMCs treatment alone (P < 0.05) (**Figures 5A, B**). Mice administered hPBMCs in combination with Dara treatment showed superior survival rates compared to mice receiving hPBMCs alone or Dara treatment alone (**Figure 5C**). Importantly, mice receiving hPBMC alone displayed a GVL effect; however, the majority of mice showed significant weight loss and ultimately died of GVHD (**Figures 5D, E**). Compared with the high GVHD scores in mice receiving hPBMC alone, no GVHD and no significant weight loss were detected in mice receiving hPBMCs in combination with Dara, demonstrating that Dara not only alleviates GVHD, but also does not abrogate the capacity of immune cells to clear tumor cells (GVL effect) (**Figures 5D, E**).

Consistent with the results from the xeno-GVHD model, Dara also induced Th17, Th1/17 and Tc1/17 proliferation in the humanized NSG-leukemia model. The frequencies of Th17 (Dara: 0.47% ± 0.08% vs Control: 0.17% ± 0.14%; P < 0.0001), Th1/17 (Dara: 4.26% ± 0.8% vs Control: 1.39% ± 0.25%; P < 0.0001) and Tc1/17 (Dara: 0.41% ± 0.13% vs Control: 0.19% ± 0.13%; P = 0.012) were significantly higher in human T cells engrafted in splenocytes from mice administered hPBMCs in combination with Dara treatment than those in control mice (**Figure 5F**).

## Discussion

CD38 is expressed not only on plasma cells but also on various immune effector cells in response to stimulation by cytokines, endotoxins and interferon, and plays a key role in the inflammatory response (7). CD38 is also expressed on Treg cells and regulatory B cells. The administration of Dara has been shown to reduce CD38-positive immune suppressor cells, including Tregs, regulatory B cells and myeloid-derived suppressor cells. Several recent reports have demonstrated that in Dara-treated multiple myeloma patients, Tregs are reduced, while helper and cytotoxic T cells are increased (5, 23, 24), which arouses concern about the theoretical risk of inducing GVHD flare-up following the use of Dara. The paradoxical roles of CD38 as a modulator of immune effector cells and immune suppressor cells prompted us to evaluate the potential immunomodulatory effects of Dara monotherapy on GVHD.

Our study is the first study to date to directly assess the impact of Dara on GVHD in a xeno-GVHD model, in which human donor T cells react against murine MHC molecules. The results of our study suggest three important characteristics of Dara that contribute to the alleviation of GVHD. First, the xeno-GVHD model provides valuable support for the further clinical use of Dara in the prophylaxis and/or treatment for GVHD. We use a humanized mouse model of GVHD in which xeno-GVHD is induced by hPBMCs transplanted into NSG mice. The model has been established to study novel drugs or regulatory T cells with wide activity in prophylaxis and the treatment of clinical GVHD (25–29), which has many advantages in comparison with classical mouse-to-mouse models of GVHD including the use of human cells to induce (and control) GVHD and the genetic diversity of immune effector cells in hPBMCs.

Furthermore, we revealed multiple mechanisms that contribute toward explaining Dara mitigation of GVHD. Firstly, Dara mainly inhibits the proliferation, activation and differentiation of CD8^+^ cytotoxic T cells. Although CD38 expression is used as a marker of T cell activation, the primary role of CD38 in immunity is not completely understood. A previous study suggested that CD38^+^CD4^+^ T cells play a myriad of roles in acute and chronic infections, which may rely on their cytotoxicity (30). Khandelwal et al. reported that peripheral blood absolute CD38^bright^CD8^+^ effector memory T (TEM) cell population expansion predicted the development of aGVHD in a prospective pilot study of 47 consecutive pediatric allo-HCT recipients (31). They further revealed that CD38^bright^CD8^+^ T cells associated with the development of aGVHD are activated, proliferating, cytotoxic trafficking cells that do not appear to respond to cytomegalovirus or Epstein–Barr virus reactivation, which suggests that CD38 is a good marker for specifically identifying T cells directly participated in aGVHD (32). Our results from preclinical xenogeneic models suggested that Dara, a CD38-directed therapeutic intervention, can prevent GVHD by inhibiting the proliferation, activation and differentiation of CD8^+^ cytotoxic T cells, which is line with previous studies and further confirms that specific activation of CD38^bright^CD8^+^ T cells is associated with the development of aGVHD.

Secondly, our study describes the previously unknown immunomodulatory effects of Dara in the mitigation of GVHD by the reduced expression of cytotoxic effector molecules (Granzyme A, Granzyme B, Granulysin, Perforin 1), pro-inflammatory cytokines (IL15, IL32, IFNγ, IL6, IL10), chemokines and chemoattractant receptors (CX3CR1, CXCR3, CCR1, CCR2, CCR5, CCL3, CCL4, CCL5) by T cells. CD38 plays dual roles as a receptor and ectoenzyme and regulates activities related to signaling and cell homeostasis. CD38 has multifunctional enzymatic activity, as both a NAD^+^ glycohydrolase (NADase) and ADP-ribosyl cyclase, and appears to be an essential modulator of intracellular NAD^+^ levels and generates a key signaling mediator, cyclic ADP-ribose (cADPR), in T cells concomitantly. T cell activation, proliferation and differentiation may be regulated by the CD38-dependent cADPR-ryanodine receptor (RyR) axis owing to its ability to modulate intracellular Ca2^+^ signaling (7). Consistent to the established role of CD38 as a T cell immune-metabolic modulator, our results of intracellular metabolic detection in human T cells after being cultured in the presence of Dara revealed that Dara modulated metabolic pathways of T cells involving purine metabolism, oxidative phosphorylation, nicotinate and nicotinamide metabolism, *etc.* Furthermore, emerging studies are indicating that the CD38-NAD^+^ axis and CD38-cADPR-RyR-Ca2^+^ signaling influence chromatin remodeling and play a key regulatory role in immune-related gene expression to determine the generation of an inflammatory versus an immunosuppressive T cell response (22). Activation of the CD38-cADPR-RyR-Ca2^+^ axis in human T cells results in nuclear localization of nuclear factor of activated T-cells cytoplasmic 1 (NFATc1) that drives the expression of several genes associated with T cell functionality, including the expression of various cytokines genes (33, 34). Transcriptome analysis revealed diminished RNA levels of numerous genes in NFATc1^˗/˗^ CD8^+^ T cells, including *TBX21*, GZMB and genes encoding cytokines and chemokines (34). Furthermore, CD38, by virtue of its NADase activity, has been shown to perturb the cellular homeostasis of two important NAD^+^ consuming enzymes, poly (ADP-ribose) polymerase (PARP) and sirtuins (Sirt), which are reported to act as epigenetic modifiers of the expression of multiple genes including cytokine and chemokine genes, and hence can alter the functional fate of T cells (7, 35–37). Consistent with the above mentioned research result, our transcriptome sequencing data of sorted human CD3^+^ T cells engrafted into the spleens obtained from Dara-treated xeno-GVHD mice also displayed changed transcription levels of RYR1, PARP1, EZH2. These compelling immunomodulatory effects of CD38, mediated *via* the regulation of immune-related gene expression, are in agreement with our observation that CD38 inhibition by Dara resulted in the reduced expression of cytotoxic effector molecules, pro-inflammatory cytokines, chemokines and chemoattractant receptors by T cells.

Intriguingly, we also observed the promotion of immunosuppressive T (Treg and Tfr) cells in response to Dara. Krejcik et al. identified a novel subpopulation of Treg cells expressing CD38 in the peripheral blood of patients with relapsed/refractory multiple myeloma, which was more immunosuppressive *in vitro* than CD38-negative Tregs and was reduced in Dara-treated patients (24). Our results suggested that Dara treatment may induce an increase in CD38-negative regulatory T cells. These conflicting data suggest the complex role of CD38 in regulating T cell responses through intervention in multiple cellular and molecular pathways. The CD38/NAD^+^/Sirt axis can impart a distinctive epigenetic signature on T cells through regulating the activity of epigenetic enzymes. Sirt1 in T cells is capable of deacetylating enhancer of zeste homolog 2 (Ezh2) and rendering it inactive; Ezh2 is an enzyme that catalyzes the methylation of H3K27 to cause transcriptional repression. Ezh2-mediated H3K27 tri-methylation is also reported to regulate the transcriptional activity of FoxP3, the signature transcription factor of Treg and Tfr cells (38–40).

Finally, and most importantly, we investigated whether Dara can separate the GVL effects from GVHD, which would impact on the potential success of applying Dara to the prophylaxis and treatment of GVHD in clinic. Using a humanized mouse model of leukemia, the highest survival rates were achieved in mice treated with hPBMCs in combination with Dara. This approach allowed for efficient GVHD prevention by Dara without loss of the GVL effects induced by hPBMCs. Our results suggested that Dara may induce Th17 (IFNγ^˗^IL17A^+^CD4^+^), Th1/17 (IFNγ^+^IL17A^+^CD4^+^), Tc17 (IFNγ^˗^IL17A^+^CD8^+^) and Tc1/17 (IFNγ^+^IL17A^+^CD8^+^) proliferation by the induction of increased IL17 mRNA expression in T cells in the xeno-GVHD model. We further confirmed that Dara also induced Th17, Th1/17 and Tc1/17 proliferation in the humanized NSG-leukemia model, which may be involved in the Dara-associated GVL effects. Chatterjee et al. reported a strategy in which Th1 and Th17 cells are merged into hybrid Th1/17 cells, which rely on glutamine-driven oxidative phosphorylation (glutaminolysis). Th1/17 cells exhibit higher levels of NAD^+^ and reduced CD38 expression. T cells with reduced surface expression of the NADase CD38 exhibited intrinsically higher NAD^+^, enhanced oxidative phosphorylation, higher glutaminolysis and altered mitochondrial dynamics that vastly improved tumor control. CD38 inhibition led to metabolic reprograming of T cells with superior tumor control (41).

In conclusion, in the current study, our findings indicate that Dara might be a promising option for separating GVHD from GVL effects in patients with hematopoietic malignancies receiving allo-HCT. It is conceivable that as anti-CD38 therapies become more available, they may present an attractive therapeutic option for future consideration.

## Data Availability Statement

The datasets presented in this study can be found in online repositories. The names of the repository/repositories and accession number(s) can be found below: NCBI SRA BioProject, accession no: PRJNA770187.

## Ethics Statement

The studies involving human participants were reviewed and approved by the Ethics Review Committee of Sir Run Run Shaw Hospital of Zhejiang University School of Medicine (Hangzhou, Zhejiang, China). The patients/participants provided their written informed consent to participate in this study. The animal study was reviewed and approved by the Ethics Review Committee of Sir Run Run Shaw Hospital of Zhejiang University School of Medicine (Hangzhou, Zhejiang, China). Written informed consent was obtained from the owners for the participation of their animals in this study.

## Author Contributions

HX designed the research study. YG, WS, TG, YW, XL, XZ, HZ, and ZC performed the experiments. YG and HX analyzed the data. YG wrote the paper and HX revised the paper. All authors contributed to the article and approved the submitted version.

## Funding

This work was supported by the National Natural Science Foundation of China (No. 81870136).

## Conflict of Interest

The authors declare that the research was conducted in the absence of any commercial or financial relationships that could be construed as a potential conflict of interest.

## Publisher’s Note

All claims expressed in this article are solely those of the authors and do not necessarily represent those of their affiliated organizations, or those of the publisher, the editors and the reviewers. Any product that may be evaluated in this article, or claim that may be made by its manufacturer, is not guaranteed or endorsed by the publisher.

## Abbreviations

GVHD, graft-versus-host disease; allo-HCT, allogeneic hematopoietic cell transplantation; GVL, graft-versus-leukemia; Dara, Daratumumab; NAD, nicotinamide dinucleotide; ADPR, adenosine 59-diphosphate-ribose; xeno-GVHD, xenogeneic GVHD; hPBMC, human peripheral blood mononuclear cell; B-NSG/B-NDG, NOD-Prkdc^scid^/IL2rg^tm1^/Bcgen; qPCR, quantitative PCR; FACS, fluorescence-activated cell sorting; FPKM, fragments per kilobase of exon per million fragments mapped; T_CM,_ central memory T cells; T_EM,_ effector memory T cells; T_Eff_, terminally differentiated effector T cells; Treg, regulatory T cells; Tfr, follicular regulatory T cells; IFNγ, interferon-gamma; TBX21, T-box transcription factor 21; RUNX3, RUNX family transcription factor 3; EOMES, eomesodermin; KLRK1, killer cell lectin like receptor K1; NKG7, natural killer cell granule protein 7; LAMP1, lysosomal-associated membrane protein 1; PECAM1, platelet and endothelial cell adhesion molecule 1; ITGA4, integrin subunit alpha 4; BTLA, B and T lymphocyte associated; RyR, ryanodine receptor; NFATc1, nuclear factor of activated T-cells cytoplasmic 1; PARP, poly (ADP-ribose) polymerase; EZH2, enhancer of zeste homolog 2.
