# Supplementary material for: Daratumumab Prevents Experimental Xenogeneic Graft-Versus-Host Disease by Skewing Proportions of T Cell Functional Subsets and Inhibiting T Cell Activation and Migration
Source: Front Immunol. 2021 Dec 20;12:785774. doi: 10.3389/fimmu.2021.785774 (PMC8720868; doi:10.3389/fimmu.2021.785774)
Supplement: Supplementary file 1 [file DataSheet_1.pdf]

Daratumumab Prevents Experimental Xenogeneic Graft-versus-host Disease by Skewing Proportions of T Cell Functional Subsets and Inhibiting T cell Activation and Migration

Yang Gao[1#], Wei Shan[2,3,4,5#], Tianning Gu[2,3,4,5#], Jie Zhang[1], Yibo Wu[2,3,4,5], Xiaoqing Li[2,3,4,5], Xiangjun Zeng[2,3,4,5], Hongyu Zhou[1], Zhi Chen[1], Haowen Xiao[1,4*]

[1]Department of Hematology, Sir Run Run Shaw Hospital, Zhejiang University School of Medicine, Zhejiang province, P R China.

[2]Bone Marrow Transplantation Center, The First Affiliated Hospital, Zhejiang University School of Medicine, Hangzhou, Zhejiang province, P R China.

[3]Liangzhu Laboratory, Zhejiang University Medical Center, Hangzhou, Zhejiang province, P R China.

[4]Institute of Hematology, Zhejiang University, Hangzhou, Zhejiang province, P R China.

[5]Zhejiang Province Engineering Laboratory for Stem Cell and Immunity Therapy, Hangzhou, Zhejiang province, P R China.

[#]Statement of equal authors' contribution: Y. Gao, W. Shan and T. Gu contributed equally to this manuscript.

***Correspondence to:** **Haowen Xiao**, M.D., Ph.D., Department of Hematology, Sir Run Run Shaw Hospital, Zhejiang University School of Medicine, No. 3 Qingchun

East Rd., Hangzhou, 310016, Zhejiang province, P R China.

Tel: +86 571 86006936; Fax: +86 571 86006936.

E-mail: haowenxiaoxiao@ zju.edu. cn

Supplementary Figures

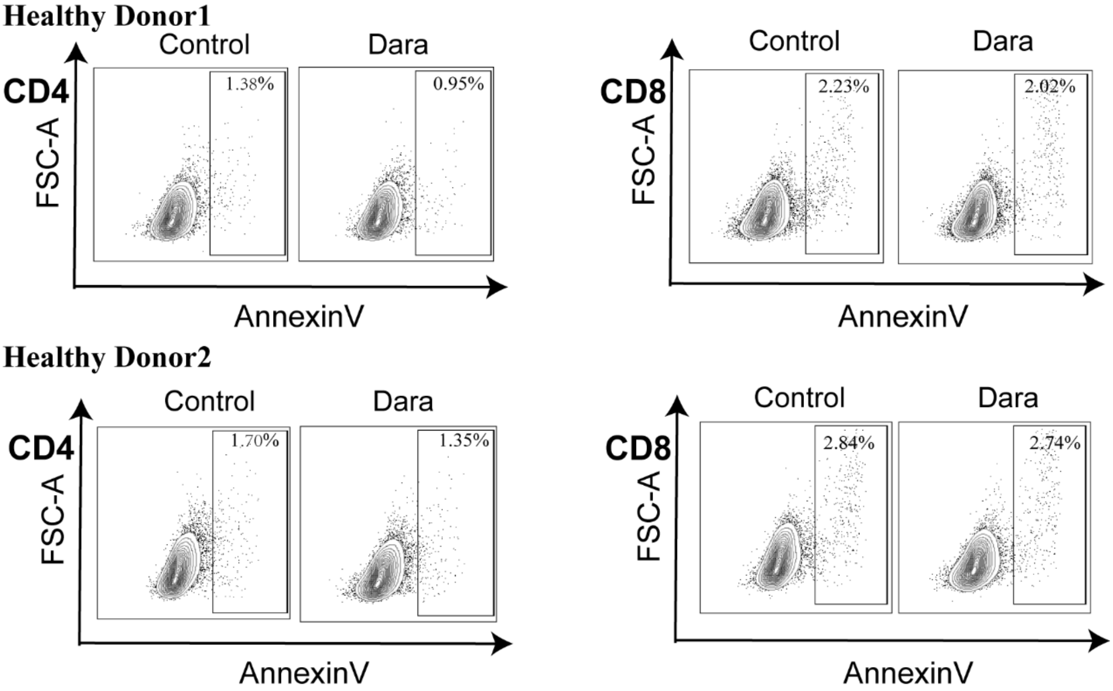

Figure S1. Human T cells were cultured in the presence or absence of Dara at 50ug/ml for 48h. Dara had no significant effect of induction of apoptosis on T cells, in which the apoptosis rates were only around 1-2% after culture with Dara. Control: in the presence of human IgG control antibody.

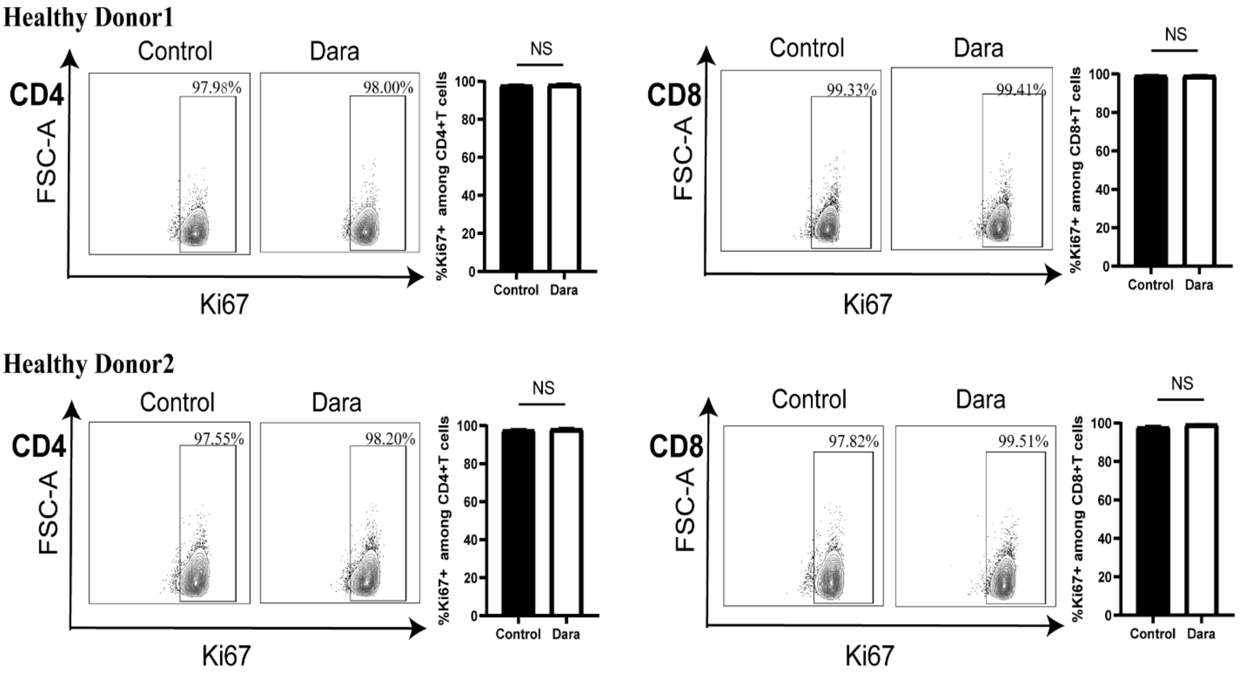

Figure S2. Human T cells were cultured in the presence or absence of Dara at 50ug/ml for 48h.There is also no significant inhibition either on CD8$^+$ T cell proliferation or on CD4$^+$ T cell proliferation. The expression levels of Ki67 on CD4$^+$ T cells (T cells from healthy donor1: Dara: 98.27% ±0.25% vs Control: 97.97% ±0.21%, P = 0.19; T cells from healthy donor2: Dara: 97.97% ±0.68% vs Control: 97.23% ±0.23%, P = 0.47) and CD8$^+$ T cells (T cells from healthy donor1: Dara: 99.3% ±0.1% vs Control: 99.27% ±0.06%, P =0.64; T cells from healthy donor2: Dara: 99.07% ±0.59% vs Control: 98.3% ±0.5%, P = 0.16) were comparable in Dara-treated T cells and control T cells.

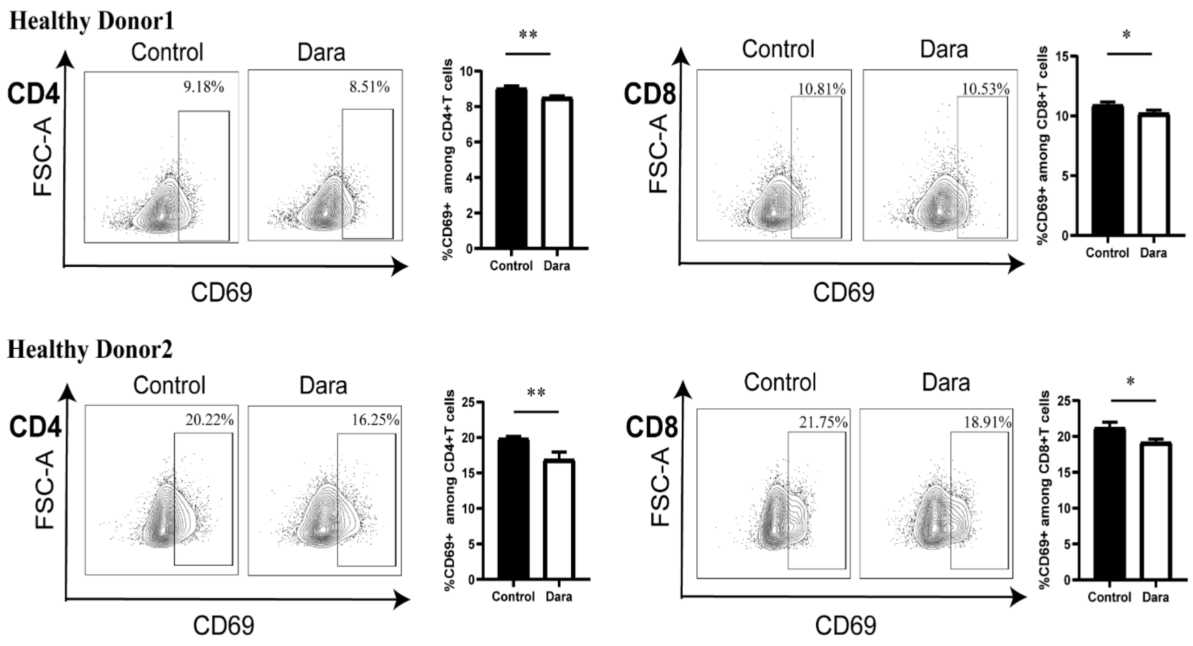

Figure S3. Human T cells were cultured in the presence or absence of Dara at 50ug/ml for 48h. A significantly decreased frequencies of CD69⁺ cells in both CD4⁺ T cells (T cells from healthy donor1: Dara: 16.90% ±1.04% vs Control: 19.87% ±0.29%, P = 0.009; T cells from healthy donor2: Dara: 8.52% ±0.09% vs Control: 9.03% ±0.13%, P = 0.0055) and CD8⁺ T cells (T cells from healthy donor1: Dara: 19.17% ±0.46% vs Control: 21.2% ±0.78%, P = 0.0178; T cells from healthy donor2: Dara: 10.22% ± 0.27% vs Control: 10.9% ±0.26%, P = 0.0358) were observed in Dara-treated T cells.

Healthy Donor1

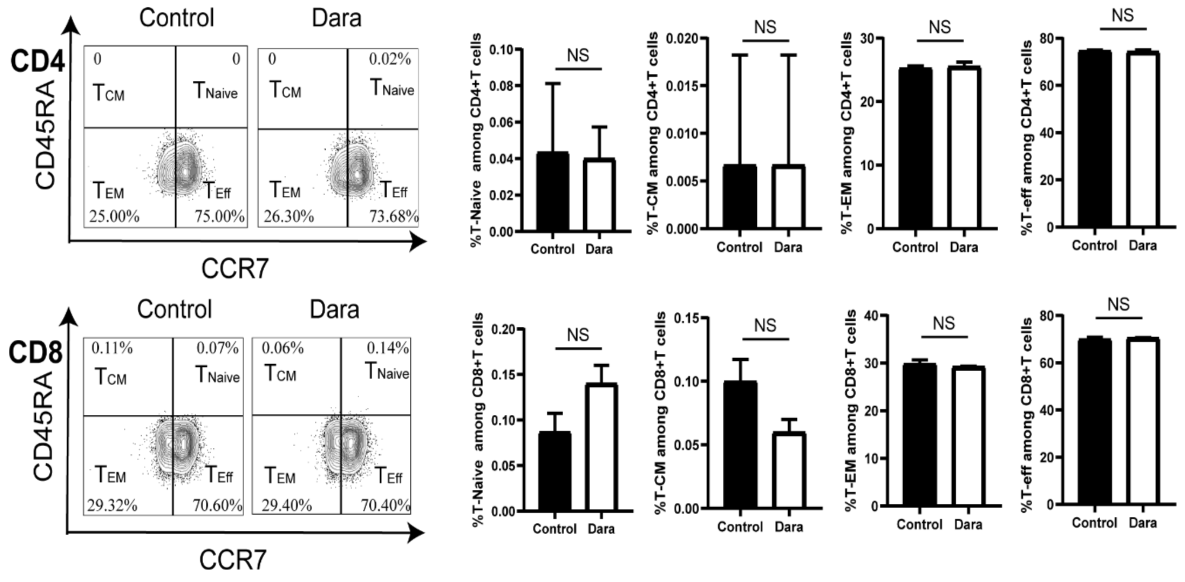

Healthy Donor2

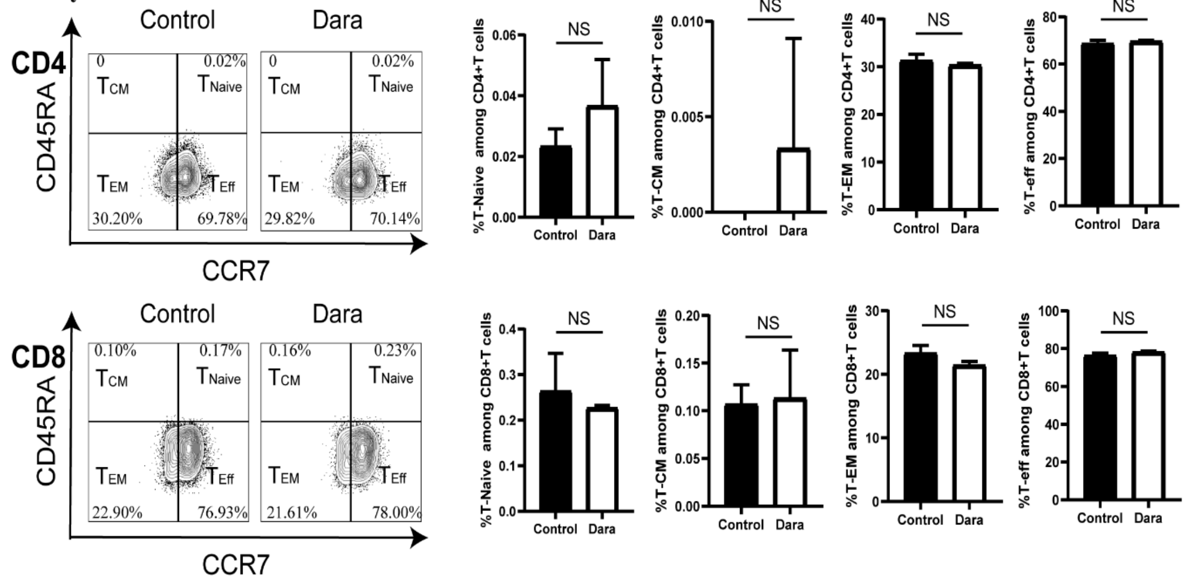

Figure S4. Human T cells were cultured in the presence or absence of Dara at 50ug/ml for 48h. Dara did not exert any inhibitory effect either on $CD4^+$ T cell differentiation or on $CD8^+$ T cell differentiation in vitro culture, with no significant increase in the frequencies of naïve/memory phenotypes.

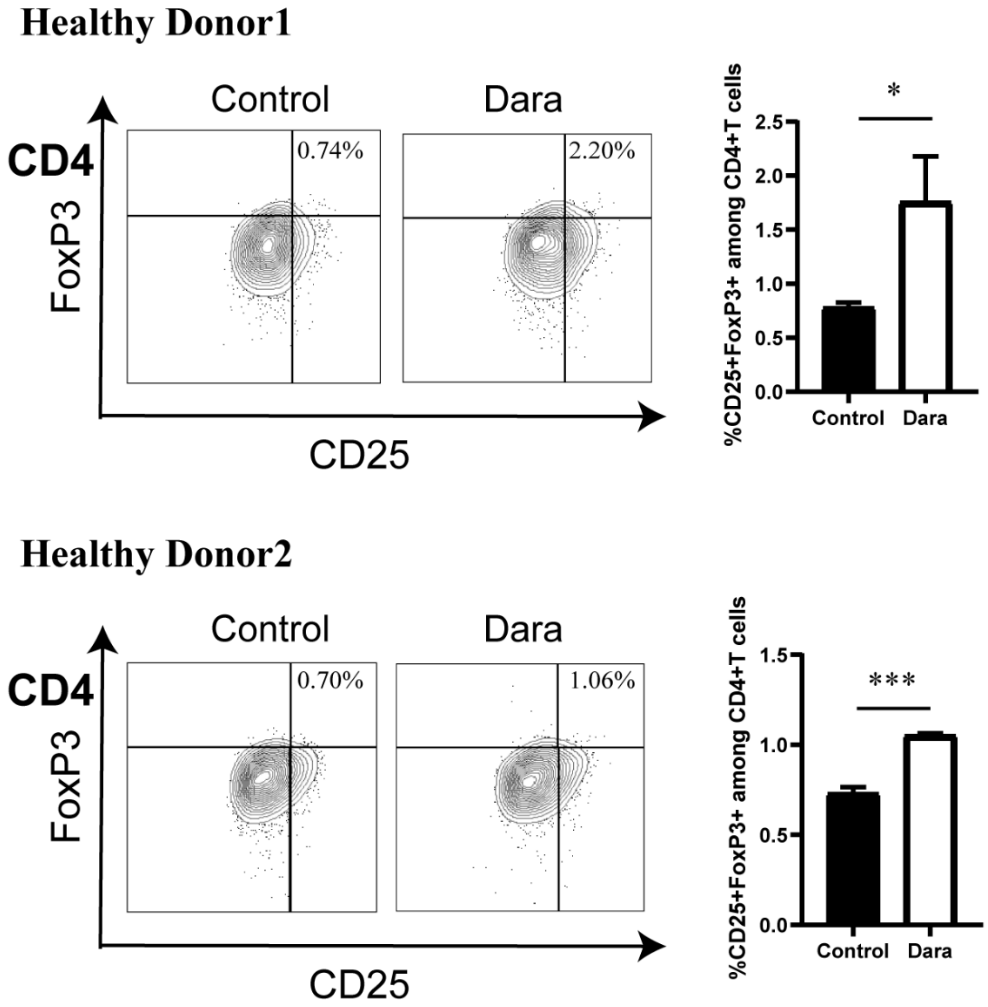

Figure S5. Human T cells were cultured in the presence or absence of Dara at 50ug/ml for 48h. The frequency of Treg (T cells from healthy donor1: Dara: 1.76% ± 0.42% vs Control: 0.78% ± 0.05%, P = 0.0163; T cells from healthy donor2: Dara: 1.05% ± 0.01% vs Control: 0.73% ±0.04%, P = 0.0001) was significantly higher in Dara-treated T cells than those from control T cells.
